# DNA Vaccine-Encoded Flagellin Can Be Used as an Adjuvant Scaffold to Augment HIV-1 gp41 Membrane Proximal External Region Immunogenicity

**DOI:** 10.3390/v10030100

**Published:** 2018-02-27

**Authors:** Lara Ajamian, Luca Melnychuk, Patrick Jean-Pierre, Gerasimos J. Zaharatos

**Affiliations:** 1 Lady Davis Institute for Medical Research, Jewish General Hospital, Montréal, QC H3T 1E2, Canada; lara.ajamian@mail.mcgill.ca (L.A.); luca.melnychuk@mail.mcgill.ca (L.M.); patjp2106@gmail.com (P.J.-P.); 2 Division of Experimental Medicine, Department of Medicine, McGill University, Montréal, QC H4A 3J1, Canada; 3 Division of Infectious Disease, Department of Medicine & Division of Medical Microbiology, Department of Clinical Laboratory Medicine, Jewish General Hospital, Montréal, QC H3T 1E2, Canada

**Keywords:** HIV-1, gp41, membrane proximal external region, MPER, flagellin, adjuvant, DNA vaccine

## Abstract

Flagellin’s potential as a vaccine adjuvant has been increasingly explored over the last three decades. Monomeric flagellin proteins are the only known agonists of Toll-like receptor 5 (TLR5). This interaction evokes a pro-inflammatory state that impacts upon both innate and adaptive immunity. While pathogen associated molecular patterns (PAMPs) like flagellin have been used as stand-alone adjuvants that are co-delivered with antigen, some investigators have demonstrated a distinct advantage to incorporating antigen epitopes within the structure of flagellin itself. This approach has been particularly effective in enhancing humoral immune responses. We sought to use flagellin as both scaffold and adjuvant for HIV gp41 with the aim of eliciting antibodies to the membrane proximal external region (MPER). Accordingly, we devised a straightforward step-wise approach to select flagellin-antigen fusion proteins for gene-based vaccine development. Using plasmid DNA vector-based expression in mammalian cells, we demonstrate robust expression of codon-optimized full length and hypervariable region-deleted constructs of *Salmonella enterica* subsp. *enterica* serovar *Typhi* flagellin (FliC). An HIV gp41 derived sequence including the MPER (gp41_607–683_) was incorporated into various positions of these constructs and the expressed fusion proteins were screened for effective secretion, TLR5 agonist activity and adequate MPER antigenicity. We show that incorporation of gp41_607–683_ into a FliC-based scaffold significantly augments gp41_607–683_ immunogenicity in a TLR5 dependent manner and elicits modest MPER-specific humoral responses in a mouse model.

## 1. Introduction

Despite the tremendous success generated by an empiric approach to vaccine development, eliciting robust and long-lasting protective immunity to certain pathogens remains challenging [1,2]. While live attenuated virus vaccines have remarkable efficacy, this is not a solution for all pathogens either because they cannot be safely attenuated or because natural infection does not confer protective immunity [2,3,4,5,6,7,8,9]. Purified protein or recombinant subunit vaccines have provided a way forward in some instances, however the immunogenicity of such antigens is often poor or vaccination simply does not generate the type of immune response required for protection [10,11]. In some instances, the magnitude, longevity or quality of the immune response to such antigens has been improved by using adjuvants, however the mechanism of adjuvant function has been ill-defined for most adjuvants [10,11].

The discovery of pathogen associated molecular patterns (PAMPs), particularly ligands for Toll-like receptors (TLRs) have revolutionized our understanding of innate immunity and ushered in a new era of rational adjuvant development [10,12,13]. One promising avenue of adjuvant research has stemmed from work with the Toll-like receptor 5 (TLR5) ligand flagellin. Polymerized flagellin proteins are the major component of the flagellar apparatus of motile gram-negative and gram-positive bacteria [14]. Monomeric flagellin proteins are the only known agonists of TLR5 [15] and a large body of work has explored the interaction between the major *Salmonella* flagellin protein, FliC and TLR5 [16,17,18,19,20,21,22]. TLR5 is expressed on a variety of cells including epithelial cells, monocytes and dendritic cells [21,23]. Flagellin interacts with TLR5 on the cell surface in a 2:2 complex and induces dimerization of flagellin-TLR5 pairs [24,25]. The resulting dimerization of the intracellular TIR domains of TLR5 activate downstream signaling pathways. This includes a MyD88-dependent signalling cascade that culminates in the induction of transcription factors, including NF-κB [16,17,18,19,20,21,22,26]. In turn, these transcription factors upregulate cytokine transcription and ultimately evoke a pro-inflammatory state.

Flagellin that gains access to the cytosol is also a trigger for the NAIP–NLRC4 inflammasome, where flagellin is sensed by the cytosolic receptor, NAIP5/6 and provokes its interaction with the adapter protein, NLRC4 [27,28,29,30,31,32,33,34,35]. This cascade triggers inflammasome assembly and subsequent caspase-1 activation [32,34,35,36,37]. Activated caspase-1 processes several pro-inflammatory cytokines including pro-IL1β and pro-IL-18, thus promoting secretion of their biologically active forms.

**Figure 1:**
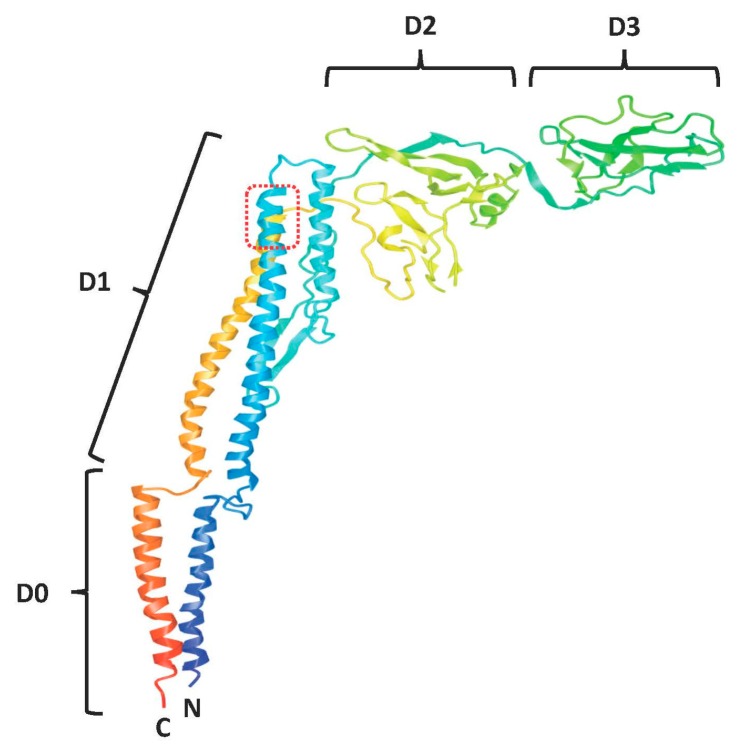
Domain structure of full-length flagellin. Image derived from PDB ID: 1UCU [38] visualized with iCn3D (https://www.ncbi.nlm.nih.gov/Structure/icn3d/full.html) and modified to indicate D0, D1, D2 and D3 domains. Residues 89–96 (QRVRELAV) in the D1 domain are encircled by a red dashed line.

Flagellin has a four-domain structure [14,21,38], wherein domains D0 and D1 are formed by interaction between the N-terminal and C-terminal portions of the protein thus producing a stalk-like structure with a concave surface. The central domains D2 and D3 form a cluster emanating at an angle from this stalk (Figure 1). The N and C-termini are critical to flagellin polymerization and certain residue stretches are highly conserved among β and γ *Proteobacteria*, whereas the D2 and D3 domains are highly variable among different bacteria [21,39]. Conserved stretches of the D1 domain contains the key residues engaging with TLR5 and remain sequestered from immune selective pressure while flagellin is in a polymerized state [24,40,41,42,43]. The C-terminal portion of the D0 domain is also highly conserved and is essential for NAIP-NLRC4 inflammasome triggering [27,32,44]. Conversely, the hypervariable region is exposed on the outside surface of the flagellar apparatus and is a dominant target for humoral responses [42,45,46,47].

Stemming from initial observations of broad and protective immune responses to live attenuated *Salmonella* oral vaccines [48], it was postulated that such vaccines might prove useful as carriers for heterologous antigens [49,50,51]. Thereafter, the insertion of heterologous epitopes into flagellin to produce a combined antigen-adjuvant module was described in seminal work using either live attenuated *Salmonella* or purified flagellin as the epitope carrier [52,53,54,55,56,57,58,59,60,61]. Stemming from these initial observations, additional studies have further defined the mechanisms behind the adjuvant effect of flagellin [21,23] and led to testing of novel vaccine designs in pre-clinical [62,63,64,65,66,67,68,69,70,71,72,73,74,75,76,77,78,79,80] and clinical studies [81,82,83,84,85]. Some have used flagellin as a stand-alone adjuvant that is concurrently delivered with antigen [86,87,88,89], whereas other studies have demonstrated a distinct advantage to incorporating heterologous epitopes within the structure of flagellin itself at either the N or C-terminus or within the hypervariable region [72,73]. Flagellin mediated enhancement of humoral responses appears to require stimulation of TLR5-expressing dendritic cells with subsequent activation of antigen-specific CD4 T-cells [21,23,26,73,86,90,91]. The TLR5-MyD88 or NAIP-NLRC4 pathway dependence of these adjuvant effects has clearly been demonstrated [21,23,33,72,91] but some work has raised the possibility that flagellin may also enhance immune responses via alternate mechanisms [92,93,94,95].

For the majority of licensed vaccines, prevention of infection correlates with the induction of pathogen-specific antibodies [96,97]. In the context of viral infection, neutralizing antibodies block interaction of the virus with target receptors on host cells and prevent entry and thus subsequent replication. Despite the recent discovery of a great number of potent HIV-1 envelope-specific broadly neutralizing antibodies, an inability to elicit such antibodies through immunization continues to hinder the HIV vaccine discovery enterprise [98,99].

The HIV gp41 membrane proximal external region (MPER) represents one important but formidable target for HIV-1 vaccine development. The MPER is a highly conserved region targeted by broadly neutralizing antibodies (bnAbs) [100]. Although such MPER-specific antibodies have been shown to prevent infection through passive immunization [101,102,103], numerous animal studies have failed to elicit robust or sufficiently broad neutralizing antibody responses using a variety of strategies [104,105,106,107,108,109,110,111,112,113,114]. Issues hindering the development of gp41 MPER as a vaccine target include poor immunogenicity due in part to steric hindrance and lack of accessibility [100,115], hydrophobicity that renders the MPER prone to aggregation in solution [116], immunodominance of adjacent gp41 regions lacking any neutralizing epitopes [117,118] and the apparent auto-reactivity of MPER-specific bnAbs towards cell membrane lipids [119,120].

Considering these challenges, we sought to use flagellin as both scaffold and adjuvant for HIV gp41, with the aim of eliciting antibodies to the MPER. We chose to pursue a gene-based vaccine approach, aiming to provide future capacity to both develop platforms that could express proteins with mammalian glycosylation and conduct iterative research with different DNA and viral vector platforms, two parameters which will likely be key to developing a protective HIV vaccine. We devised a straightforward step-wise approach to select FliC-antigen fusion proteins for gene-based vaccine development. Accordingly, we generated plasmid DNA vaccine vectors encoding a variety of FliC-HIV gp41 fusion proteins and screened candidate vaccines based on adequate mammalian cell expression, fusion protein secretion, TLR5 agonist activity and gp41 MPER antigenicity. Insertion of a gp41-derived sequence at different positions within FliC led to a broad spectrum of outcomes with regard to secretion and agonist activity. Utilizing this multi-modal process, we selected a promising vaccine candidate that fulfilled all our screening criteria. We demonstrate that this FliC-HIV gp41 DNA vaccine candidate was highly immunogenic relative to a DNA vaccine encoding gp41 alone, eliciting modest MPER-specific humoral immunogenicity. Moreover, the augmented immunogenicity of the vaccine was TLR5-dependant.

## 2. Materials and Methods

### 2.1. Construction of FliC and HIV-1 gp41 Expression Vectors

All flagellin (FliC) and gp41 gene constructs described were codon optimized and synthesized using the GENEART platform (http://www.lifetechnologies.com/ca/en/home/life-science/cloning/gene-synthesis/geneart-gene-synthesis/geneoptimizer.html) (Life Technologies, Carlsbad, CA, USA). To produce a mammalian cell expressed FliC, we used an amino acid sequence identical to that of *Salmonella enterica* subsp. *enterica* serovar *Typhi* flagellin (GenBank: AAA27067.1). Numbering of FliC residues in our constructs was based on a previously established numbering convention [18]. We previously described generating a Clade C gp41 ectodomain consensus sequence [121]. Based on this sequence we subsequently selected a shorter sequence corresponding to the gp41 ectodomain spanning amino acids 607 to 683 in gp160 (according to HxB2 numbering), herein referred to as gp41_607–683_ (see Supplementary Methods 1). For all gene constructs, a tPA signal sequence [122] was added synthetically to the N-terminus and a GGGS linker and 3XFLAG tag were added to the C-terminus. For constructs used to produce coating antigen for antibody binding assays, a Twin-Strep-Tag was also added C-terminal to the 3XFLAG tag. Synthesized genes were cloned into the pVAX plasmid using BamHI and NotI enzymes. Deletion and point mutants and fusion constructs were generated using the Seamless Cloning Kit (Life Technologies, Carlsbad, CA, USA). The following constructs were created: gp41_607–671_ (lacking the twelve most C-terminal residues making up the MPER), FliC R90D, FliC Δ89–96, FliC Δ174–400, FliC Δ220–320, FliC Δ89–96 Δ174–400, FliC Δ89–96 Δ220–320, gp41_607–683_ FliC Δ174–400, gp41_607–683_ FliC Δ220–320, FliC Δ174-[gp41_607–683_]-400, FliC Δ220-[gp41_607–683_]-320, FliC gp41_607–683_, FliC Δ174–400 gp41_607–683_, FliC Δ220–320 gp41_607–683_ and FliC Δ89–96 Δ174–400 gp41_607–683_. All plasmid DNA expression vectors used in downstream transfection or vaccination experiments were prepared using a Qiagen EndoFree Plasmid kit (Qiagen, Hamburg, Germany).

### 2.2. Cell Culture

HEK 293T cells (CRL-3216; ATCC, Manassas, VA, USA) and the mouse monocyte/macrophage cell line J774A.1 (TIB-67; ATCC, Manassas, VA, USA) were each cultured in Dulbecco’s Minimal Essential Media (DMEM) supplemented with 10% Fetal Bovine Serum (FBS), 10 mM HEPES, 100 U/mL penicillin and 100 μg/mL streptomycin (all from Corning, Tewksbury, MA, USA). The human monocyte cell line, THP-1 (TIB-202; ATCC, Manassas, VA, USA) was cultured in RPMI-1640 and the same supplements as well as 0.05 mM 2-mercaptoethanol (Sigma, St Louis, MO, USA). HEK-Blue-hTLR5 cells (Invivogen, San Diego, CA, USA) were cultured in Dulbecco’s Minimal Essential Media (DMEM) supplemented with 10% FBS, 10 mM HEPES, 50 U/mL penicillin, 50 μg/mL streptomycin, 30 μg/mL blasticidin and 100 μg/mL zeocin (the latter two reagents from Invivogen, San Diego, CA, USA). All cells were cultured at 37 °C and 5% CO_2_ in a humidified incubator.

### 2.3. Transfection and Cell Lysate/Supernatant Preparation

HEK 293T cells were seeded at 3 × 10^6^ cells/well in a 6 well plate 24 h prior to transfection. JetPEI (Polyplus, Illkirch, France) was used for transient transfection of 293T cells. Media was changed 24 h after transfection to DMEM supplemented with 1% FBS but otherwise constituted as above. Cell lysates and supernatants were collected 48 h after transfection. Cells were washed twice with phosphate-buffered saline (PBS) and spun down at 300 g for 10 min. Cells were lysed in NP40 cell lysis buffer (Life Technologies, Carlsbad, CA, USA) containing a protease inhibitor cocktail (Sigma, St Louis, MO, USA). Cells supernatants were clarified by centrifugation for 15 min at 16,000 g. Cell lysates and clarified supernatants were stored at −20 °C until analyzed.

### 2.4. SDS-PAGE, Immunoblotting and Deglycosylation Studies

All SDS-PAGE and immunoblotting reagents and devices were from the same manufacturer (Life Technologies, Carlsbad, CA, USA), unless otherwise stated. All procedures were carried out as previously described [121]. Cell lysates and supernatants were incubated at 70 °C for 10 min after the addition of 4X LDS Loading Buffer and Reducing Agent. Samples were run on NuPAGE 4–12% Bis-Tris gels at 150 V under reducing conditions. Gels were transferred to PVDF membranes using the iBlot transfer device. Membranes were blocked for 20 min in 10% non-fat dry milk dissolved in Tris-buffered saline containing 0.05% Tween (TBS-T). Resolved proteins were detected by blotting with a mouse anti-FLAG tag antibody, clone M2 (Sigma, St. Louis, MO, USA) diluted to 0.5 μg/mL in 3% milk TBS-T or a mouse anti-Tubulin antibody, clone B3 (Thermo Scientific, Waltham, MA, USA) diluted to 1:2500 in 1% milk TBS-T. A secondary goat anti-mouse IgG antibody conjugated to alkaline phosphatase (AP) was diluted to 1:5000 in TBS-T and used to detect the primary antibody. CDP-star substrate was used to generate a chemiluminescent signal, which was detected using X-ray film (VWR, Radnor, PA, USA) and an X-ray imager (Kodak, Rochester, NY, USA). In deglycosylation studies, we used PNGase F (NEB, Ipswich, MA, USA) to remove N-glycans from proteins of interest, secreted into cell supernatants. Cell supernatants at a volume of 13.5 μL were incubated with 1.5 μL of glycoprotein denaturing buffer (NEB, Ipswich, MA, USA) for 10 min at 99 °C. Samples were then treated with 1 μL PNGase F for 1 h at 37 °C in the presence of 2 μL NP-40 and 2 μL G7 buffer (NEB, Ipswich, MA, USA).

### 2.5. Secretion ELISA

Cell supernatants (100 μL per well) were applied to anti-FLAG tag antibody-coated plates (GenScript, Piscataway, NJ, USA) and incubated for 1 h at room temperature (RT). Plates were subsequently washed three times with PBS-T, then incubated with a mouse anti-FLAG tag antibody conjugated to horseradish peroxidase (HRP) (Sigma, St. Louis, MO, USA) at a 1:20,000 dilution for 1 h at RT. After incubation, plates were washed three times and relative amounts of secreted protein were detected using the QuantaRed substrate system (Thermo Scientific, Waltham, MA, USA). Data were collected as relative fluorescence units (RFU) using a fluorescence plate reader with an excitation/emission filter of 544 nm/612 nm. The relative level of secreted FLAG-tagged protein determined by this secretion ELISA was used to normalize input for the TLR5 agonist activity and antibody binding assays described below.

### 2.6. TLR5 Agonist Activity Assays

#### 2.6.1. HEK-Blue-hTLR5

A cell suspension of 140,000 cells/mL of HEK-Blue-hTLR5 was prepared and 180 μL was plated in each well of a 96-well plate containing 20 μL of a 1:100 dilution of previously normalized supernatants containing flagellin proteins. HEK-Blue-hTLR5 cell suspensions used were produced by scraping and dissociation by gentle pipetting. Cell culture supernatants were normalized based on the relative amounts of secreted protein determined by the secretion ELISA (described above). Treated HEK-Blue-hTLR5 cells were incubated for 20 h prior to determining the quantity of secreted embryonic alkaline phosphatase (SEAP) released into the supernatant using the QUANTI-Blue colorimetric enzyme assay (Invivogen, San Diego, CA, USA) as per manufacturer’s instructions. Briefly, 5 μL of treated HEK-Blue-hTLR5 supernatant from each condition was added to 200 μL of QUANTI-Blue reagent. After one hour incubation at 37 °C, SEAP was quantified by measuring absorbance at 655 nm. Relative quantities, presented as response ratios, were calculated by dividing the absorbance of each condition by the absorbance of the control condition.

#### 2.6.2. Transcriptional Activation of IL-1β in Monocyte/Macrophage Cell Lines

A cell suspension of 140,000 cells/mL of either THP-1 or J774A.1 cells was prepared then 180 μL was transferred to wells of a 96-well plate. Subsequently, 20 μL of a 1:100 dilution of previously normalized supernatants containing flagellin proteins was added to each well. J774A.1 cell suspensions used were produced by scraping and dissociation by gentle pipetting. LPS (Invivogen, San Diego, CA, USA) diluted in culture medium at a final concentration of 1 μg/mL was used as a positive control. Empty vector-transfected cell supernatant was used as a negative control. Treated cells were incubated for 2 h then harvested and RNA was extracted using an RNeasy mini kit (Qiagen, Valencia, CA, USA) with on-column DNase digestion as per the manufacturer’s instructions. Eluted RNA was used in a two-step RT-PCR reaction, utilizing the SuperScript IV First-Strand Synthesis System and Platinum Hot Start PCR Master Mix (both from Life Technologies, Carlsbad, CA, USA) as per the manufacturer’s instructions. Random hexamers were used in reverse transcription and the following primers were used for PCR: il1beta forward 5′-TGTAATGAAAGACGGCACACC-3′; il1beta reverse 5′-TCTTCTTTGGGTATTGCTTGG-3′; gapdh forward 5′-AGCTTGTCATCAACGGGAAG-3′; gapdh reverse 5′-TTTGATGTTAGTGGGGTCTCG-3′. RT-PCR products underwent agarose gel electrophoresis and were stained with SYBR Safe DNA Gel Stain (Life Technologies, Carlsbad, CA, USA) then visualized with a blue-light transilluminator.

### 2.7. ELISA to Detect Binding by Monoclonal bnAb

To assess bnAb binding to secreted gp41_607–683_ or FliC gp41_607–683_ proteins, cell supernatants were diluted in DMEM according to the relative level of gp41 determined by the secretion ELISA as previously described [121], then plated on streptavidin coated plates (Thermo Scientific, Waltham, MA, USA) for 1 h at RT and subsequently washed three times with PBS-T. Each gp41 antigen was synthetically linked to a 3XFLAG and Twin-Strep-Tag at its C-terminus. The following HIV-1 gp41 monoclonal antibody reagents were generously provided by investigators via the NIH AIDS Reagent Program, Division of AIDS, NIAID, NIH: 4E10 (from Dr. Hermann Katinger) and 10E8 (from Dr. Mark Connors). Each bnAb was diluted to 1 μg/mL in 100 μL of PBS-T containing 5% bovine serum albumin (BSA), then applied to wells and allowed to bind for 1 h at RT. Plates were washed three times and bnAb binding was probed using a mouse anti-human IgG AP-conjugated antibody (Life Technologies, Carlsbad, CA, USA) diluted 1:5000 in PBS-T containing 5% BSA for 1 h at RT. Plates were then washed three times, AttoPhos fluorescent substrate (Promega, Fitchburg, WI, USA) was applied to wells and the plate was incubated for 15 min at RT. Data were collected as relative fluorescence units (RFU) using a fluorescence plate reader with an excitation/emission filter of 485 nm/520 nm. Results were normalized to levels of secreted protein captured in each well using an HRP-conjugated anti-FLAG tag antibody and QuantaRed substrate as described above.

### 2.8. Mice and Immunizations

All procedures were approved by the institutional Animal Care Committee of McGill University (Protocol #2012-7237 JGH; first approval date: 01/01/2013). Guidelines and regulations established by the Canadian Council on Animal Care were adhered to and all experiments complied with ARRIVE guidelines. Six-week-old female BALB/c mice were obtained from Charles River Laboratories (Montréal, QC, Canada) and housed under pathogen-free conditions. Mice were injected with 50 μg of DNA in each *tibialis anterior* muscle (50 μL at a concentration of 1 μg/μL in sterile normal saline) with either empty vector negative control (pVAX) or one of three DNA vaccines (gp41_607–683_, FliC Δ174–400 gp41_607–683_ or FliC Δ89–96 Δ174–400 gp41_607–683_). Mice were immunized with DNA vaccines either two or four times, depending on the experiment, at two week intervals. Blood was collected 2 weeks after the last immunization. Whole blood was allowed to clot for 1 h at room temperature then spun down at 2000 g for 15 min. Sera were then collected and stored at −80 °C until analysis.

### 2.9. ELISA to Detect Mouse Anti-gp41 IgG Response

Streptavidin-coated (Roche, Basel, Switzerland) or Strep-Tactin-coated plates (IBA Life Sciences, Goettingen, Germany) were coated with either gp41_607–683_ or gp41_607–671_ overnight at 4 °C. Each gp41 antigen was synthetically linked to a 3XFLAG and Twin-Strep-Tag at its C-terminus. Antigen was coated onto plates from clarified supernatants diluted to a concentration of 2 μg/μL of the protein of interest. The concentration was determined by ELISA and a standard of FLAG tag immunoprecipitation-purified gp41 as previously described [121]. Coated plates were washed three times with PBS-T containing 0.01% BSA. Serum from each mouse was serially diluted up to 1:200 (see Supplementary Methods 2) in PBS-T containing 0.01% BSA, applied to the plates and incubated for 2 h at room temperature. For experiments assessing MPER specificity of humoral response, sera from the FliCΔ174–400 gp41_607–683_ vaccinated group were pooled prior to dilution and processed as described above. Plates were washed three times and antibody binding was probed using a goat anti-mouse IgG AP-conjugated antibody (Life Technologies, Carlsbad, CA, USA) diluted to 1:5000 in PBS-T containing 0.01% BSA. Plates were incubated for 1 h, then washed three times. The bound AP-conjugated secondary antibody was detected using the ELISA Amplification System (Life Technologies, Carlsbad, CA, USA) as per manufacturer’s instruction. Plates were read on an ELISA plate reader at an absorbance of 495 nm. An independent two sample t-test was used to determine statistical significance between experimental arms.

## 3. Results

### 3.1. Codon-Optimized Salmonella enterica Subsp. enterica Serovar Typhi FliC Can Be Expressed in and Effectively Secreted from Mammalian Cells

**Figure 2:**
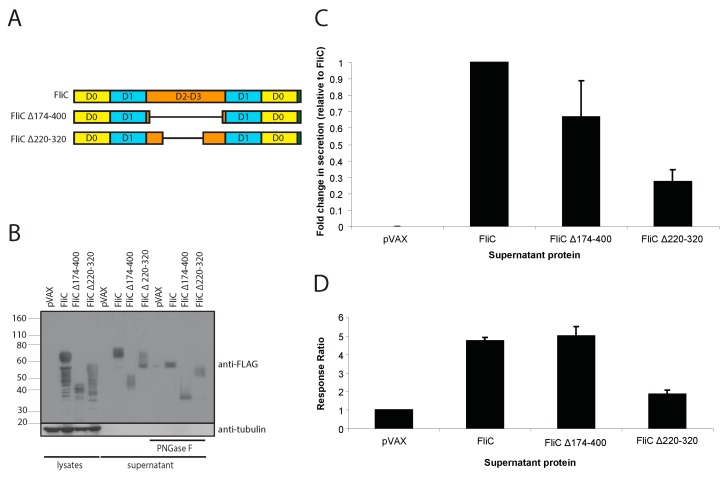
FliC constructs expressed in a mammalian system are secreted and maintain Toll-like receptor 5 (TLR5) agonist activity. (**A**) Schematic representation of flagellin (FliC) constructs; (**B**) Western blot of cell lysates, supernatants and PNGase F treated supernatants from transiently transfected 293T. Cells were transfected with pVAX (empty vector), FliC, FliC Δ174–400 or FliC Δ220–320. Samples were collected 48 h post transfection. Blots were probed with a mouse anti-FLAG tag antibody to detect FLAG-tagged flagellin proteins. A mouse anti-tubulin antibody, was used to detect tubulin as a loading control; (**C**) Measurement of relative secretion level of FLAG-tagged proteins using capture ELISA. Supernatants from transiently transfected 293T were applied to anti-FLAG tag antibody coated plates and probed with a horseradish peroxidase (HRP) conjugated mouse anti-FLAG tag antibody. Results shown represent the mean of 4 different experiments where FliC is set to a value of 1. Error bars represent standard error of the mean; (**D**) Relative TLR5 agonist activity of secreted FliC proteins. Normalized supernatants from transiently transfected 293T were diluted 1:100 and added to HEK-Blue-hTLR5 cells. After 20 h incubation, the quantity of secreted embryonic alkaline phosphatase (SEAP) produced was determined using a colorimetric enzyme assay. Relative quantities, presented as response ratios, are indicative of TLR5 agonist activity. Results shown represent the mean of 4 different experiments where pVAX is set to a value of 1. Error bars represent standard error of the mean.

We first set out to determine if the FliC gene could be well expressed and effectively secreted by plasmid DNA vector-transfected cells. We produced mammalian codon-optimized FliC and inserted it into a CMV promoter driven expression plasmid (pVAX). As the hypervariable region of FliC has been shown to be a dominant antigenic region [42,47] that is not necessary for TLR5 agonist function, we also generated 2 hypervariable region deletion mutants of FliC, one lacking residues 174–400 (FliC Δ174–400) and another lacking residues 220–320 (FliC Δ220–320) (Figure 2A) and inserted them into the same backbone vector. Similar deletion mutants have previously been described [42,47]. After transfection of 293T cells, we gauged the level of expression and secretion using both immunoblotting and ELISA-based methods. We observed robust expression of our three FliC variants in cell lysates (Figure 2B), however we also visualized a ladder of products migrating below the predicted size of protein variants suggesting that a portion of the expressed proteins underwent proteolytic cleavage. Moreover, while the predicted molecular weight for full length FliC is approximately 60 kDa, the largest and most abundant protein observed in our lysates migrated at approximately 80 kDa. Similar results were observed for our deletion mutants (FliC Δ174–400 and FliC Δ220–320), with the largest band migrating at a higher molecular mass then predicted. In corresponding supernatants, protein bands at or above the predicted molecular weight were also observed (Figure 2B). These findings suggested that the FliC variant, particularly the secreted forms, had undergone extensive glycosylation. Indeed, flagellin is glycosylated in bacterial systems [123]. It has also been previously shown that baculovirus encoded FliC undergoes N-linked glycosylation when expressed in insect cells (*Spodoptera frugiperda* Sf9) [78]. In silico analysis revealed that the FliC amino acid sequence we utilized contains at least four asparagine residues predicted to be N-glycosylated (http://www.cbs.dtu.dk/services/NetNGlyc/ and Figure S1). To address this possibility, supernatants were treated with PNGase F to remove N-linked glycosylations. Treatment with PNGase F resulted in the disappearance of the higher molecular mass proteins and visualization of proteins of the predicted size for all three FliC variants (Figure 2B), thus confirming that FliC is glycosylated when expressed in a mammalian system. A shift in molecular weight upon PNGase treatment was also observed in cell lysates for all three proteins (Figure S2). Of the three FliC variants, full length FliC appeared to be the most highly expressed and secreted (Figure 2B). To better estimate the relative degree of secretion of each variant, an ELISA-based assay was used to measure the FLAG-tagged secreted proteins in culture supernatants. All FliC variants were readily detectable, however relative to full length FliC, FliCΔ174–400 and especially FliCΔ220–320 exhibited diminished secretion (Figure 2C).

### 3.2. Mammalian Cell-Expressed and Secreted FliC Retains TLR5 Agonist Activity

Next, we sought to ascertain whether mammalian expressed FliC maintained TLR5 agonist activity. To this end, we applied transfected cell culture supernatants containing either full length FliC, FliC Δ174–400 or FliC Δ220–320 to HEK-Blue-hTLR5 cells, a TLR5 agonist activity indicator cell. Full length FliC and FliC Δ174–400 demonstrated robust TLR5 agonist activity, however FliC Δ220–320 only elicited a minor response (Figure 2D).

**Figure 3:**
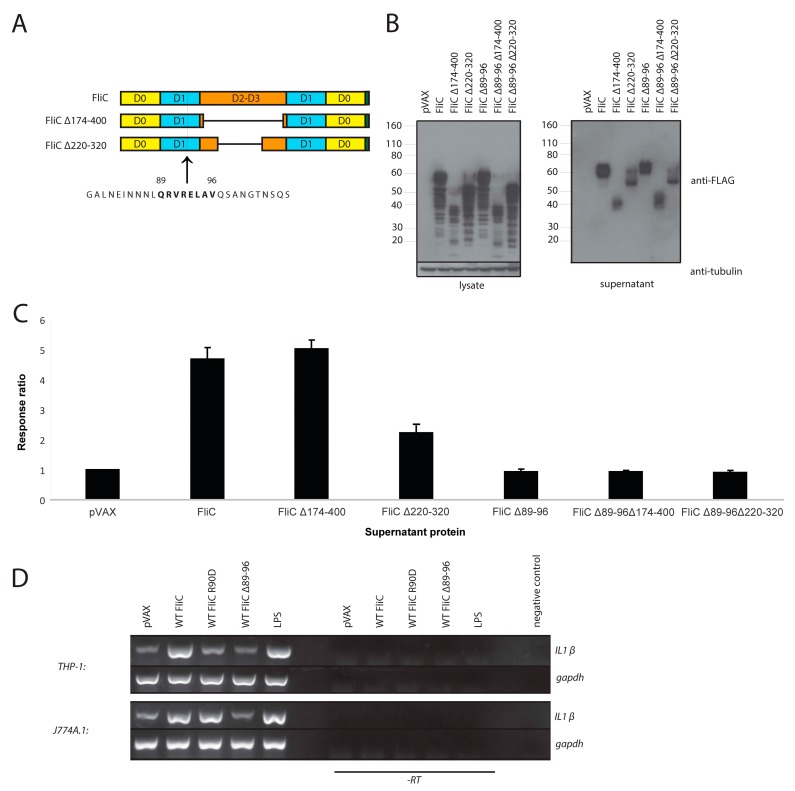
Residues QRVRELAV (89–96) of FliC are required for TLR5 agonist activity. (**A**) Schematic representation of FliC constructs; (**B**) Western blots of cell lysates and supernatants from transiently transfected 293T. Cells were transfected with pVAX, FliC, FliC Δ174–400, FliC Δ220–320, FliC Δ89–96, FliC Δ89–96 Δ174–400 or FliC Δ89–96 Δ220–320. Blots were probed with a mouse anti-FLAG tag antibody to detect FLAG-tagged flagellin proteins. A mouse anti-tubulin antibody, was used to detect tubulin as a loading control; (**C**) Relative TLR5 agonist activity of secreted FliC proteins. Normalized supernatants from transiently transfected 293T were diluted 1:100 and added to HEK-Blue-hTLR5 cells. After 20 h incubation, the quantity of SEAP produced was determined using a colorimetric enzyme assay. Relative quantities, presented as response ratios, are indicative of TLR5 agonist activity. Results shown represent the mean of 4 different experiments where pVAX is set to a value of 1. Error bars represent standard error of the mean; (**D**) Induction of IL-1β transcription detected by RT-PCR and gel electrophoresis. Normalized supernatants from transiently transfected 293T were diluted 1:100 and added to THP-1 or J774A.1 cells. Cells were incubated for 2 h then harvested and RNA was obtained for RT-PCR with IL-1β specific primers. Cells treated with LPS or with supernatant from empty vector transfected cells were used as induced and non-induced controls, respectively. RT-PCR with GAPDH specific primers was used as an internal control. To exclude potential DNA carry-over and amplification or contamination, control reactions omitting the RT enzyme (-RT) or omitting input RNA (negative control) are also shown.

To further confirm that our FliC variants were indeed signaling through TLR5, we sought to modify residues in FliC that are necessary for TLR5 agonist activity. Residues 89–96 (QRVRELAV), in the D1 domain of FliC, are required for FliC TLR5 agonist function [41,42]. We therefore produced deletion mutants of this region (Δ89–96) in full length FliC, FliC Δ174–400 and FliC Δ220–320 (Figure 3A). All Δ89–96 constructs were expressed and secreted to a similar extent as their parent constructs (Figure 3B). No TLR5 agonist activity was observed with any construct containing the Δ89–96 deletion as compared to their parental constructs and in fact their responses were no different than that produced by the empty vector control construct (Figure 3C). This confirmed that the responses produced by the indicator cell line upon exposure to FliC, FliC Δ174–400 and FliC Δ220–320 were dependent on the TLR5 agonist activity of FliC.

Having confirmed TLR5 agonist activity for our FliC variants using HEK-Blue-hTLR5 cells, we then wished to gauge their capacity for triggering a signaling cascade via TLR5 in a more biologically relevant system. To this end we used monocyte cell lines THP-1 (human) and J774A.1 (mouse). These cell lines express TLR5 on their surface and engagement of TLR5 by flagellin would activate signaling pathways that would ultimately lead to the transcriptional upregulation of pro-inflammatory cytokines [124,125]. Accordingly, we exposed these cells to either full length FliC, FliC Δ89–96 or full-length FliC containing the point mutation R90D (FliC R90D). Mutations at position R90 in FliC have been previously shown to diminish or abrogate signaling via mouse or human TLR5, respectively [18,126,127,128].

Focusing on one such cytokine, IL-1β, we compared the relative abundance of IL-1β transcripts in THP-1 or J774A.1 cells after exposure to either full length FliC, FliC R90D or FliC Δ89–96. In parallel, cells were also exposed to either positive or negative control conditions using LPS or supernatant from empty vector-transfected cells, respectively. In THP-1 cells, full length FliC and LPS clearly induced IL-1β (Figure 3D). FliC R90D and FliC Δ89–96 failed to induce any IL-1β beyond the basal level present in our negative control conditions. Results with J774A.1 cells were similar except that FliC R90D retained its ability to induce IL-1β (Figure 3D). The difference in intra-species sensitivity to FliC R90 mutant agonist activity was in keeping with previously published work [126,127,128]. Overall, these experiments further confirmed that mammalian cell expressed FliC variants can trigger a signaling cascade via TLR5.

### 3.3. Adding gp41 MPER (gp41_607–683_) to the C Terminus of FliCΔ174–400 Results in a Secreted Protein that Maintains TLR5 Agonist Activity

**Figure 4:**
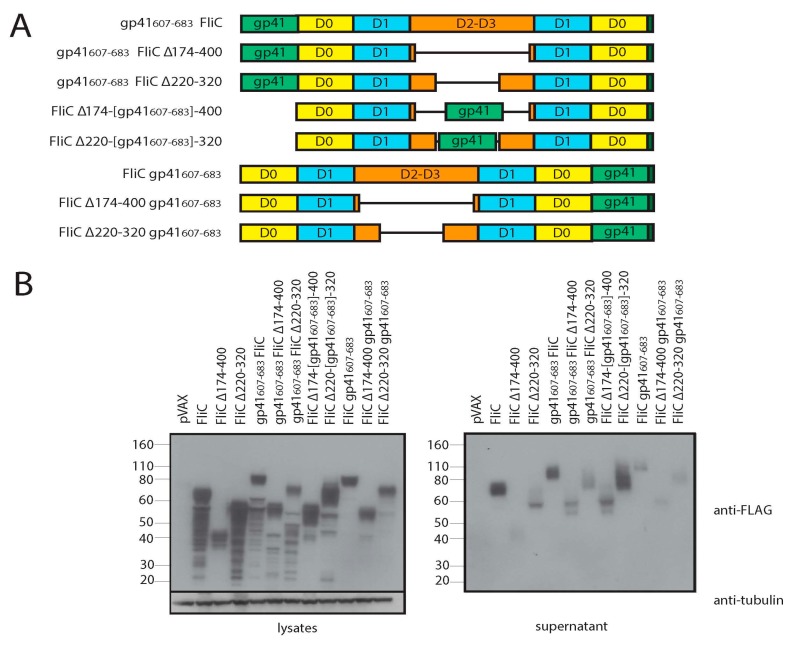

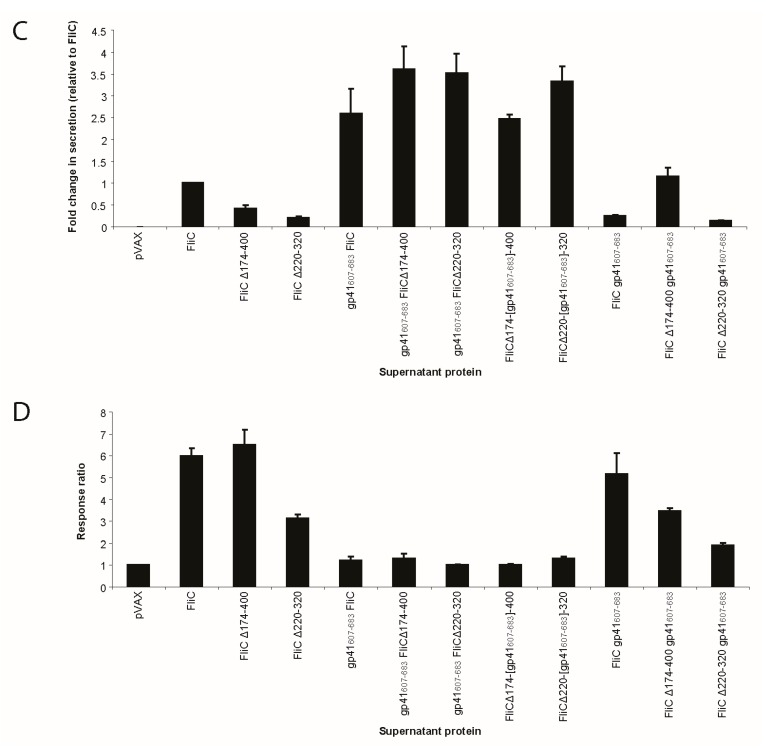
FliC Δ174-400 with gp41_607-683_ inserted at its N-terminus is secreted and maintains TLR5 agonist activity. (**A**) Schematic representation of flagellin constructs; (**B**) Western blot of cell lysates and supernatants from transiently transfected 293T. Cells were transfected with pVAX, FliC, FliC Δ174–400, FliC Δ220–320, gp41_607–683_ FliC, gp41_607–683_ FliC Δ174–400, gp41_607–683_ FliC Δ220–320, FliC Δ174-[gp41_607–683_]-400, FliC Δ220-[gp41_607–683_]-320, FliC gp41_607–683_, FliC Δ174–400 gp41_607–683_ or FliC Δ220–320 gp41_607–683_. Samples were collected 48 h post transfection. Blots were probed with a mouse anti-FLAG tag antibody to detect FLAG-tagged flagellin proteins. A mouse anti-tubulin antibody, was used to detect tubulin as a loading control; (**C**) Measurement of relative secretion level of FLAG-tagged proteins using capture ELISA. Supernatants from transiently transfected 293T were applied to anti-FLAG tag antibody coated plates and probed with an HRP conjugated mouse anti-FLAG tag antibody. Results shown represent the mean of 3 different experiments where FliC is set to a value of 1. Error bars represent standard error of the mean; (**D**) Relative TLR5 agonist activity of secreted FliC proteins. Normalized supernatants from transiently transfected 293T were diluted 1:100 and added to HEK-Blue-hTLR5 cells. After 20 h incubation, the quantity of SEAP produced was determined using a colorimetric enzyme assay. Relative quantities, presented as response ratios, are indicative of TLR5 agonist activity. Results shown represent the mean of 3 different experiments where pVAX is set to a value of 1. Error bars represent standard error of the mean.

We next sought to use FliC as both a scaffold and adjuvant for HIV gp41. To determine the optimal position to insert gp41 (amino acids 605–683, gp41_607–683_ herein), we generated eight different constructs wherein gp41_607–683_ was inserted at the amino- or carboxy-terminus of full length FliC, FliC Δ174–400 and FliC Δ220–320, or alternatively within the remaining hypervariable regions of FliC Δ174–400 and FliC Δ220–320 (Figure 4A). All proteins were expressed and secreted to varying degrees as observed by immunoblotting (Figure 4B). An ELISA-based assay was once again used to measure the secreted proteins in culture supernatants (Figure 4C). The level of secretion varied widely among the different constructs. Relative to full length FliC, gp41_607–683_ insertion at the amino-terminus or within the hypervariable region of FliC Δ174–400 and FliC Δ220–320 variants resulted in an increase in protein secretion (Figure 4C). Insertion of gp41_607–683_ at the carboxy-terminus produced variants that were poorly secreted except for the FliC Δ174–400 variant.

Having gauged the relative secretion levels of our constructs, we next sought to determine if they were able to maintain TLR5 agonist activity. Despite their superior propensity for secretion, once normalized for the relative amount of secreted protein, FliC variants with gp41_607–683_ inserted at the amino-terminus or within the hypervariable regions of FliC Δ174–400 and FliC Δ220–320, manifested weaker TLR5 agonist activity relative to their parent constructs (Figure 4D). Instead we observed that insertion of gp41_607–683_ at the carboxy-terminus preserved TLR5 agonist activity to a greater degree.

### 3.4. Adding gp41 MPER (gp41_607–683_) to the C Terminus of FliCΔ174–400 Maintains gp41 Antigenicity

**Figure 5:**
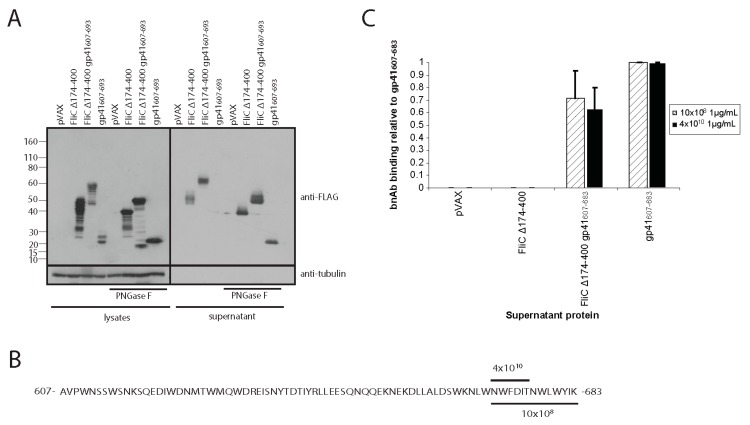
FliC Δ174–400 gp41_607–683_ and gp41_607–683_ have similar gp41 antigenicity. (**A**) Western blot of cell lysates, supernatants and PNGase F treated supernatants from transiently transfected 293T. Cells were transfected with pVAX, FliC Δ174–400, FliC Δ174–400 gp41_607–683_ or gp41_607–683_. Samples were collected 48 h post transfection. Blots were probed with a mouse anti-FLAG tag antibody to detect FLAG-tagged flagellin proteins. A mouse anti-tubulin antibody, was used to detect tubulin as a loading control; (**B**) Schematic representation of 4E10 and 10E8 binding sites; (**C**) Binding of MPER-specific broadly neutralizing antibodies (bnAB) to FliC Δ174–400 gp41_607–683_ and gp41_607–683_. Normalized supernatants from transiently transfected 293T were added to streptavidin coated plates. Captured antigen was probed with either 10E8 or 4E10 antibodies, then an AP-conjugated mouse anti-human IgG secondary antibody was used to detect antigen-bound 10E8 and 4E10. Binding was measured using a fluorescent AP substrate assay and further normalized to levels of captured antigen in each well. Results shown represent the mean of 3 different experiments where gp41_607–683_ is set to a value of 1. Error bars represent standard error of the mean.

As we wished to minimize humoral responses to the FliC hypervariable region, and FliC Δ174–400 produced superior TLR5 agonist activity compared to FliC Δ220–320 (Figure 4D), we chose to pursue the development FliC Δ174–400 gp41_607–683_ as a DNA vaccine candidate. FliC Δ174–400 gp41_607–683_, FliC Δ174–400 and gp41_607–683_ were readily detectable in transiently transfected cell lysates and treatment of lysates with PNGase allowed better visualization of each protein (Figure 5A). Immunoblotting also allowed us to readily detect secreted proteins although gp41_607–683_ was only detectable after PNGase treatment (Figure 5A). To test if FliC Δ174–400 gp41_607–683_ maintained gp41 antigenicity, we next sought to ascertain if this protein was bound by the bNAbs 10E8 and 4E10 (Figure 5B) as compared to positive (gp41_607–683_ in the absence of flagellin) and negative (FliC Δ174–400) control proteins. Our experiments revealed that FliC Δ174–400 gp41_607–683_ was bound by 10E8 and 4E10, albeit to a lesser degree than gp41_607–683_ (Figure 5C), thus demonstrating preservation of gp41 antigenicity.

### 3.5. FliC Augments gp41_607–683_ Immunogenicity and Elicits MPER-Specific Humoral Responses

**Figure 6:**
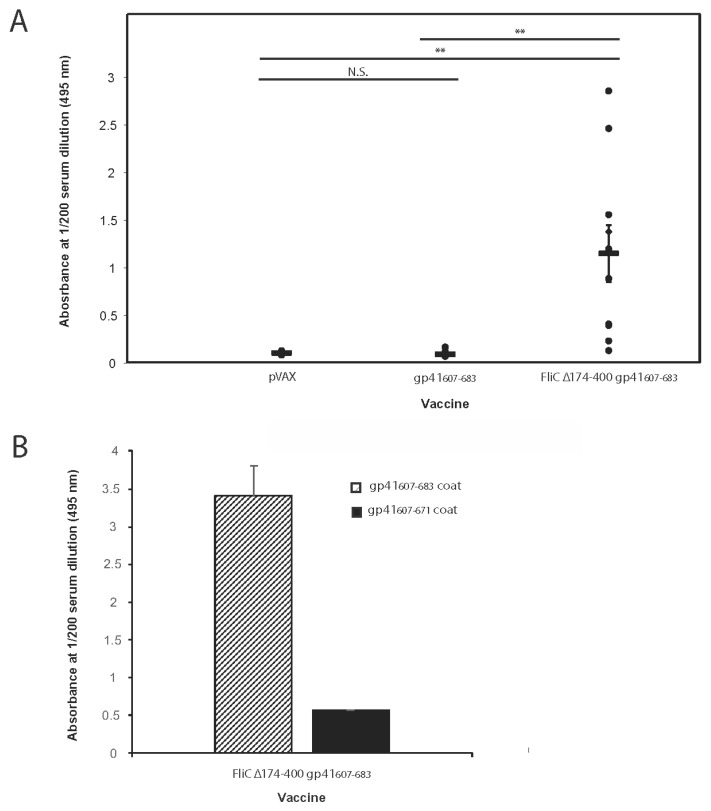
FliCΔ174–400 augments HIV-1 gp41_607–683_ immunogenicity. (**A**) Detection of HIV-1 gp41_607–683_ binding antibodies in vaccinated mice. Female BALB/c mice (10 mice per experimental arm) were injected intramuscularly with either pVAX (empty vector) or DNA vaccines FliCΔ174–400 gp41_607–683_ or gp41_607–683_ (50 μL in each hind leg at a concentration of 1 μg/μL). Two weeks following the 4th vaccination, mouse serum (1:200 dilution) was analyzed for a gp41_607–683_ specific IgG response. Each point represents the mean absorbance value obtained from individual mouse serum (each analyzed in duplicate). Horizontal bars represent average values per vaccine group and error bars represent +/− standard error of the mean. Significance was determined using an unpaired *t*-test with ** indicating a *p* value < 0.01; (**B**) Detection of antibodies binding C-terminal residues of HIV-1 gp41_607–683_. Mouse sera from the mice vaccinated with FliCΔ174–400 gp41_607–683_ were analyzed for an MPER-specific IgG response by comparing binding to gp41_607–683_ (containing the entire MPER) and gp41_607–671_ (lacking the twelve C-terminal residues of MPER). Vertical bars graphs represent the mean absorbance value obtained for pooled sera (*n* = 10 mice) analyzed in duplicate. Error bars represent standard error of the mean.

Having determined that FliC Δ174–400 gp41_607–683_ retained gp41 antigenicity, we then proceeded to gauge the immunogenicity of the expressed protein in the context of a DNA vaccine. Mice were immunized with one of three DNA vaccines: pVAX (empty vector control), gp41_607–683_ or FliC Δ174–400 gp41_607–683_. Mice were immunized on days 0, 14, 28 and 42 and blood was collected 2 weeks post the fourth immunization. We then measured gp41-specific IgG responses by ELISA (Figure 6A). For the majority of the mice who received FliC Δ174–400 gp41_607–683_, strong responses were detectable out to a 1/200 dilution of serum (Figure 6A). Although the response to FliC Δ174–400 gp41_607–683_ was modest, it was significantly greater than the response elicited by gp41_607–683_. At this dilution, sera from mice that received gp41_607–683_ alone manifested responses that were no better than sera from mice who received an empty vector control vaccine (Figure 6A).We then wished to determine if the humoral response generated by FliC Δ174–400 gp41_607–683_ targeted the C-terminal portion of the MPER or rather residues N-terminal to this region, such as the immunodominant region or the HR2 region [100]. We therefore coated our ELISA plate with either gp41_607–683_ as above or a truncated protein devoid of the majority of the MPER (gp41_607–671_), then gauged the specificity of pooled sera from our FliC Δ174–400 gp41_607–683_ vaccinated group. The response to the protein containing the MPER was at least five-fold greater in magnitude than that to the protein lacking the MPER (Figure 6B), suggesting that immunization with FliC Δ174–400 gp41 _607–683_ did indeed elicit an MPER-specific response. Taken together our results revealed that FliC, deleted of a large portion of its hypervariable region, augmented humoral responses to gp41_607–683_ and especially the MPER, when gp41_607–683_ was incorporated into its C-terminus.

### 3.6. The Adjuvant Effect of FliC is TLR5 Dependent

**Figure 7:**
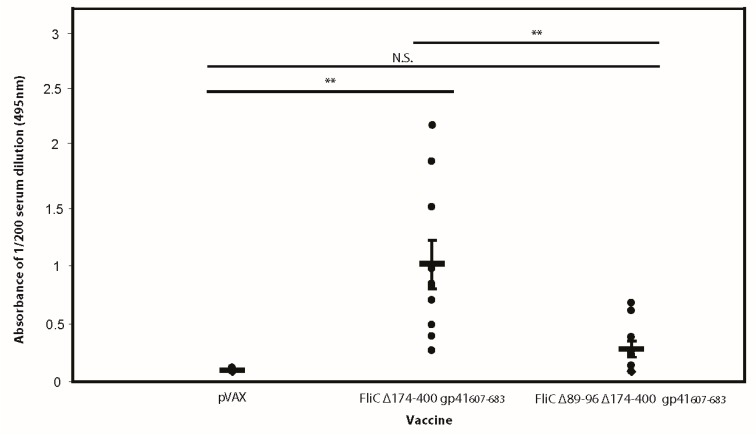
Deletion of FliC residues 89–96 results in a reduced adjuvant effect on HIV-1 gp41_607–683_ immunogenicity. Female BALB/c mice (10 mice per experimental arm) were injected intramuscularly with either pVAX (empty vector) or DNA vaccines FliCΔ174–400 gp41_607–683_ or FliC Δ89–96 Δ174–400 gp41_607–683_ (50 μL in each hind leg at a concentration of 1 μg/μL). Two weeks following the 4th vaccination, mouse serum (1:200 dilution) was analyzed for a gp41_607–683_ specific IgG response. Each point represents the mean absorbance value obtained from individual mouse serum (each with two replicates per mouse). Horizontal bars represent average values per vaccine group and error bars represent standard error of the mean. Significance was determined using an unpaired *t*-test with ** indicating a *p* value < 0.01.

Our DNA vaccine studies revealed that flagellin did indeed impart an adjuvant effect that enhanced immunogenicity of the gp41 antigen. Accordingly, we then wished to gauge the extent to which the adjuvant effect was TLR5 agonist-related or alternatively whether the flagellin scaffold merely acted as a carrier. To do so, we immunized mice with one of three vaccines: pVAX (empty vector control), FliC Δ174–400 gp41_607–683_ or FliC Δ89–96 Δ174–400 gp41_607–683_. In order to minimize any accumulated effect of CD4 helper responses on the humoral response, each group only received a vaccine on days 0 and 14. Blood was collected 2 weeks post the second immunization and analyzed for gp41-specific responses by ELISA. Relative to FliC Δ174–400 gp41_607–683_, an inferior response was observed for mice vaccinated with FliC Δ89–96 Δ174–400 gp41_607–683_ (Figure 7), indicating that the majority of the adjuvant effect of the FliC scaffold was indeed TLR5 dependent.

## 4. Discussion

The present work demonstrates that codon-optimized *Salmonella* FliC can be expressed in and effectively secreted from mammalian cells. The secreted FliC retains TLR5 agonist activity. These findings are in agreement with previous work where a gene-based or viral vector approach was used to express flagellin [127,129,130,131].

Other investigators have selected a variety of sites for insertion of heterologous into flagellin [23]. This has primarily included either the N or C-terminus [63,64,71,72,73,74,75,90] or sites within the hypervariable region [67,69] and in some instances both the hypervariable region and C-terminus were used [62,68,79,84]. Although this selection seems at times to be empiric, these sites have historically been selected to both fully expose the inserted epitopes and avoid disrupting conserved flagellin residues, particularly those amino acids shown to be required for TLR5 agonist activity. 

We postulated that the location of the inserted heterologous sequence, in our case HIV gp41_607–683_, would affect the degree of secretion and the TLR5 agonist activity of the resulting fusion protein. Indeed, upon testing we noted tremendous variation for different fusion proteins in regard to these two parameters. While some fusion proteins were highly secreted, their ability to trigger TLR5 mediated signaling was relatively poor. Accordingly, we pursued antigenicity and immunogenicity testing for FliC Δ174–400 HIV gp41_607–683_, the vaccine candidate that fulfilled both parameters and also lacked immunodominant FliC epitopes [42,45,46,47]. We subsequently showed that although FliC Δ174–400 HIV gp41_607–683_ appeared not to be as readily bound by MPER-specific bnAbs, relative to gp41_607–683_, it was markedly more immunogenic and appeared to elicit MPER-specific humoral responses. Moreover, we showed that the enhancement of immunogenicity was indeed dependent on TLR5 interaction.

We did not pursue immunization experiments in TLR5 knock out mice but instead compared humoral responses between mice that received FliC Δ174–400 HIV gp41_607–683_ to those that received FliC Δ89–96 Δ174–400 gp41_607–683_. As expected, immunization with the latter vaccine, which lacked residues essential to TLR5 interaction [41,42], produced a humoral response of relatively low magnitude that was only marginally superior to immunization with an empty vector control vaccine. Although, we have not definitively excluded a contribution of the NAIP/NLRC4 inflammasome [33] to the adjuvant effect of FliC on humoral responses in the present work, the near complete loss of adjuvant activity with FliC Δ89–96 Δ174–400 gp41_607–683_ argues against this possibility.

It could be argued that the adjuvant activity we observed was due in part to binding of our expressed fusion protein to TLR5 on dendritic cells or other APC, resulting in facilitated internalization and antigen processing rather than solely initiation of a pro-inflammatory signaling cascade [33,73,90,91,93]. Indeed, we cannot exclude the possibility that TLR5-mediated internalization by APC played some role in adjuvant activity or that antigen internalization, dendritic cell maturation and the induction of a pro-inflammatory milieu occurred contemporaneously.

Several observations in our present work generate further questions that we believe should be pursued. Two types of post-translational modification were evident in the context of our experiments. First, we discovered that vector-expressed FliC was significantly glycosylated. Indeed, this was predicted by in silico analysis. Moreover, to augment secretion of expressed FliC, all our vectors incorporated a signal peptide fused to the N-terminus of the expressed gene. This design would be expected to guide the protein to the secretory pathway where *N*-glycosylation of FliC might produce progressively oligomeric and branched structures. It is unclear if such glycosylation is advantageous. Whereas future efforts to purify fusion proteins might be complicated by the presence of a broad spectrum of glycoforms, *N*-linked glycosylation of FliC might enhance protein stability or perhaps shield and prevent unmasking of sub-dominant B-cell epitopes and thus allow the humoral response to focus on any inserted heterologous epitopes. Second, vector-expressed FliC which accumulated intracellularly appeared to undergo extensive proteolytic degradation. We did not address the mechanism of degradation in our experiments. Proteasomal or lysosomal degradation may have been induced by over-expression and protein misfolding or aggregation [132]. It is also interesting to note that the C-terminus of FliC is disordered and appears to be a target of cleavage and subsequent FliC instability [133]. Specifically addressing these issues might provide strategies to augment secretion of intact FliC and FliC-antigen fusion proteins. Conversely, some propensity for proteolytic cleavage may be advantageous if FliC is taken up by APC and more readily processed for loading onto MHC II.

A major conclusion of the present work is that the insertion site of a heterologous antigen within the FliC scaffold has a marked impact on the resulting fusion protein’s secretion and TLR5 agonist activity. It is conceivable that excessive protein aggregation might modulate these parameters. Alternatively, interference of the inserted antigen with either TLR5 binding or ligand-receptor dimerization, via inter or intramolecular interaction, might hinder robust signaling. Larger inserts might have a more dramatic impact on these parameters. Such considerations might influence the desirability of using flagellin as an epitope scaffold rather than a stand-alone co-delivered adjuvant.

We did not specifically address to what extent immune humoral responses were also generated towards the FliC scaffold itself. Although the hypervariable region we deleted is known to contain the immunodominant FliC epitopes [42,45,46,47], it is possible that its deletion allowed subdominant B-cell epitopes in other regions to elicit a greater response. This may be important to consider with further development, for several reasons. First, dominance of B-cell epitopes within FliC might detract to some extent from a robust humoral response to inserted heterologous epitopes. Second, the generation of antibodies to regions important for TLR5 binding might neutralize FliC’s potential to act as an adjuvant [42,127]. We must also consider that as FliC and gp41 are delivered together in the context of a fusion protein, FliC CD4 epitopes might provide CD4 T-cell help for antibody responses to gp41. Further assessment of this possibility should be carried out, both to delineate potential helper epitopes and to gauge the need to introduce heterologous helper epitopes to further enhance the humoral response to gp41.

While we successfully obtained humoral responses to gp41, and to some extent MPER-specificity, the magnitude of responses remained modest. Other investigators have also explored using flagellin as an adjuvant to augment responses to the HIV-1 envelope proteins [52,134,135,136] but have not explored flagellin’s potential to augment responses to gp41 MPER. Prior to expanding our research to include immunization of rabbits or guinea pigs and evaluation of HIV-1 neutralization capacity, we must both address some of the outstanding questions posed above and devise a regimen to further improve immunogenicity. Immunogenicity of our present DNA vaccine, FliC Δ174–400 HIV gp41_607–683_, would likely be significantly improved by enhanced delivery methods such as in vivo electroporation. A prime-boost strategy will also likely be necessary to achieve the desired level of immunogenicity. Accordingly, DNA prime-protein boost or DNA prime-viral vector boost regimens should be explored.

## Supplementary Materials

The following are available online at http://www.mdpi.com/1999-4915/10/3/100/s1, Supplementary Methods 1: HIV-1 gp41 sequence selection, Supplementary Methods 2: End-point titer selection, Figure S1: In silico prediction of N-glycosylation of FliC amino acid residues, Figure S2: Western blot of cell lysates from transiently transfected 293T before and after treatment with PNGase F.
