# Supplementary material for: DNA Vaccine-Encoded Flagellin Can Be Used as an Adjuvant Scaffold to Augment HIV-1 gp41 Membrane Proximal External Region Immunogenicity
Source: Viruses. 2018 Feb 27;10(3):100. doi: 10.3390/v10030100 (PMC5869493; doi:10.3390/v10030100)
Supplement: Supplementary file 1 [file viruses-10-00100-s001.zip › Flagellin_Supplementary Material_2018.02.21/Figure S1. In silico prediction of N-glycosylation of FliC amino acid residues_figure legend.docx]

**Figure S1.** ***In silico* prediction of N-glycosylation of FliC amino acid residues.** Graph indicating predicted N-glycosylation sites across the protein chain from the N- to C-terminus, with potential sites (green vertical lines) crossing the threshold (blue horizontal line at 0.5) as determined using NetNGlyc 1.0 (<http://www.cbs.dtu.dk/services/NetNGlyc/)>.
