# Supplementary material for: DNA Vaccine-Encoded Flagellin Can Be Used as an Adjuvant Scaffold to Augment HIV-1 gp41 Membrane Proximal External Region Immunogenicity
Source: Viruses. 2018 Feb 27;10(3):100. doi: 10.3390/v10030100 (PMC5869493; doi:10.3390/v10030100)
Supplement: Supplementary file 1 [file viruses-10-00100-s001.zip › Flagellin_Supplementary Material_2018.02.21/Figure S2. Western blot of cell lysates from transiently transfected 293T before and after treatment with PNGase F_figure legend.docx]

**Figure S2.** **Western blot of cell lysates from transiently transfected 293T before and after treatment with PNGase F.** Cells were transfected with FliC, FliC Δ174-400 or FliC Δ220-320. Samples were collected 48h post transfection. Blots were probed with a mouse anti-FLAG tag antibody to detect FLAG-tagged flagellin proteins.
