# Supplementary material for: DNA Vaccine-Encoded Flagellin Can Be Used as an Adjuvant Scaffold to Augment HIV-1 gp41 Membrane Proximal External Region Immunogenicity
Source: Viruses. 2018 Feb 27;10(3):100. doi: 10.3390/v10030100 (PMC5869493; doi:10.3390/v10030100)
Supplement: Supplementary file 1 [file viruses-10-00100-s001.zip › Flagellin_Supplementary Material_2018.02.21/Supplementary Methods.docx]

**Supplementary Methods**

*1. HIV-1 gp41 sequence selection*

The sequence selected (AVPWNSSWSNKSQEDIWDNMTWMQWDREISNYTDTIYRLLEESQNQQEKNEKDLLALDSWKNLWNWFDITNWLWYIK, where the MPER sequence in underlined) referred to as gp41_607-683_, was selected based on *in silico* prediction of its suitability for accessing the secretory pathway, when introduced downstream of the tPA signal sequence, using the SignalP tool (<http://www.cbs.dtu.dk/services/SignalP/)>.

*2. End-point titer selection*

The end-point titer selected (1:200) was based on preliminary experiments where this titer was the highest mean dilution of FliC Δ174-400 gp41_607-683_ vaccinated mouse sera that resulted in an optical density exceeding that of the mean of mock vaccinated mouse sera (pVAX, empty vector control) plus five times the standard deviation (SD) and where any single vaccinated mouse sera fell within one SD of the mean of mock vaccinated mouse sera.
