# Supplementary figures and images for: DNA Vaccine-Encoded Flagellin Can Be Used as an Adjuvant Scaffold to Augment HIV-1 gp41 Membrane Proximal External Region Immunogenicity
Source: Viruses. 2018 Feb 27;10(3):100. doi: 10.3390/v10030100 (PMC5869493; doi:10.3390/v10030100)

NetNGlyc 1.0: predicted N-glycosylation sites in Sequence

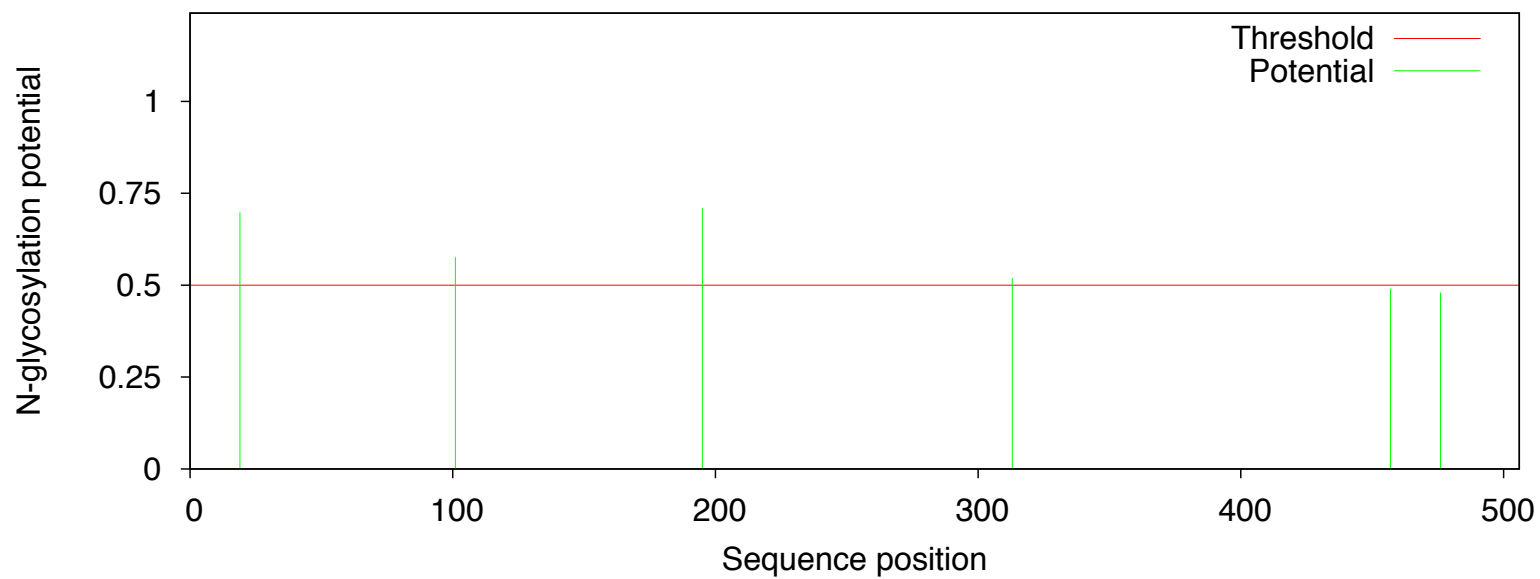

Supplement: Supplementary file 1 [file viruses-10-00100-s001.zip › Flagellin_Supplementary Material_2018.02.21/Figure S1. In silico prediction of N-glycosylation of FliC amino acid residues.pdf]

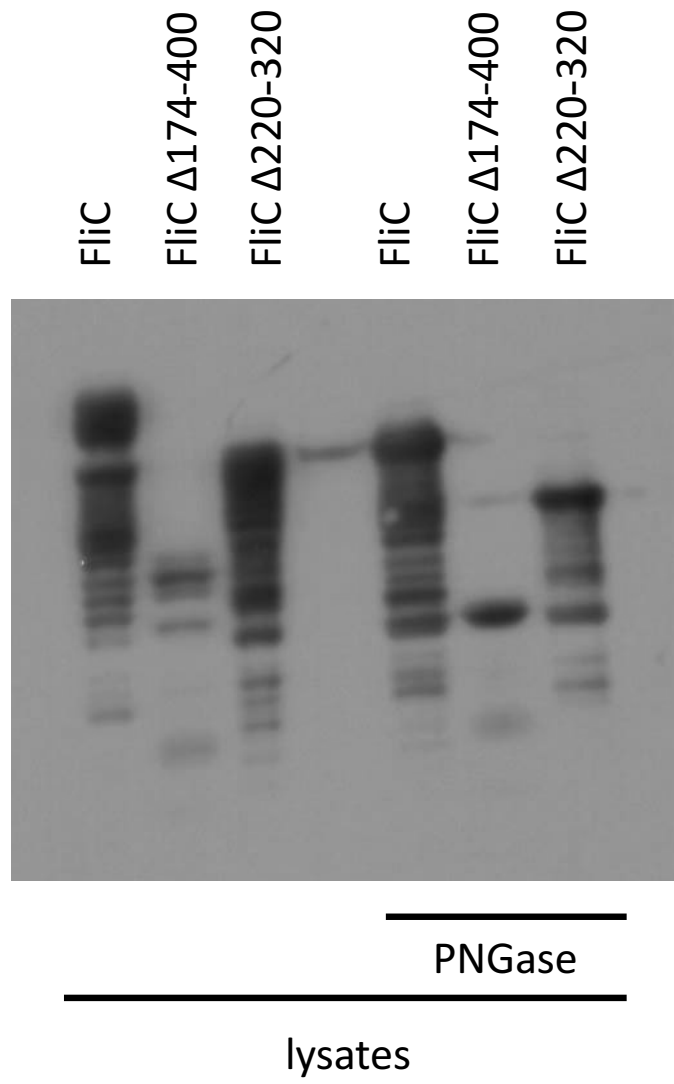

Supplement: Supplementary file 1 [file viruses-10-00100-s001.zip › Flagellin_Supplementary Material_2018.02.21/Figure S2. Western blot of cell lysates from transiently transfected 293T before and after treatment with PNGase F.pdf]
